# Focal unspecific bone uptake on [^18^F]-PSMA-1007 PET: a multicenter retrospective evaluation of the distribution, frequency, and quantitative parameters of a potential pitfall in prostate cancer imaging

**DOI:** 10.1007/s00259-021-05424-x

**Published:** 2021-06-13

**Authors:** Hannes Grünig, Alexander Maurer, Yannick Thali, Zsofia Kovacs, Klaus Strobel, Irene A. Burger, Joachim Müller

**Affiliations:** 1 Department of Nuclear Medicine, University Hospital Zurich, University of Zurich, Zurich, Switzerland; 2 Department of Nuclear Medicine and Radiology, Cantonal Hospital Lucerne, Lucerne, Switzerland; 3 Department of Radiology and Nuclear Medicine, Cantonal Hospital St. Gallen, St. Gallen, Switzerland; 4 Department of Nuclear Medicine, Cantonal Hospital Baden, Baden, Switzerland

**Keywords:** Staging, Restaging, [^18^F]-PSMA, Bone metastasis, PET/CT, PET/MR

## Abstract

**Purpose:**

Improved logistics and availability led to a rapid increase in the use of [^18^F]-PSMA-1007 for prostate cancer PET imaging. Initial data suggests increased uptake in benign lesions compared to [^68^ Ga]-PSMA-11, and clinical observations found increased unspecific bone uptake (UBU). We therefore investigate the frequency and characteristics of UBU in [^18^F]-PSMA-1007 PET.

**Methods:**

We retrospectively analyzed [^18^F]-PSMA-1007 PET scans from four centers for the presence of UBU, defined as a focal mild-to-moderate uptake (SUV_max_ < 10.0) not obviously related to a benign or malignant cause. If present, up to three leading UBUs were quantified (SUV_max_), localized, and correlated to clinical parameters, such as age, PSA, injected dose, Gleason score, tumor size (T1–T4), and type of PET scanner (analog vs. digital). Additionally, clinical and imaging follow-up results and therapeutic impact were evaluated.

**Results:**

UBUs were identified in 179 out of 348 patients (51.4%). The most frequent localizations were ribs (57.5%) and pelvis (24.8%). The frequency of UBUs was not associated with PSA, Gleason score, tumor size, age, or the injected [^18^F]-PSMA-1007 dose. UBUs were significantly more frequent in images obtained with digital PET/CT scans (n = 74, 82%) than analog PET/CT scans (n = 221, 40.3%) (*p* = .0001) but not in digital PET/MR (n = 53, 51%) (*p* = .1599). In 80 out of 179 patients (44.7%), the interpretation of UBUs was critical for therapeutic management and therefore considered clinically relevant. For 65 UBUs, follow-ups were available: three biopsies, three radiotherapies with PSA follow-up, and 59 cases with imaging. After follow-up, UBUs were still considered unclear in 28 of 65 patients (43%), benign in 28 (43%), and malignant in nine (14%) patients.

**Conclusion:**

UBUs occur in two-thirds of patients imaged with [^18^F]-PSMA-1007 PET/CT and are significantly more frequent on digital PET scanners than analog scanners. UBUs should be interpreted carefully to avoid over-staging.

**Supplementary Information:**

The online version contains supplementary material available at 10.1007/s00259-021-05424-x.

## Introduction

Positron emission tomography (PET), combined with either computer tomography (CT) or magnetic resonance imaging (MRI), utilizing radiotracers that bind to prostate-specific membrane antigen (PSMA) is an excellent diagnostic tool for prostate cancer imaging. In the past few years, PSMA-PET has evolved to become the leading advanced imaging modality, especially for patients with early biochemical recurrence (BCR) [1, 2]. In various studies, PSMA-PET/CT has shown better detection efficacy in early BCR than MRI, CT, conventional imaging [3–6], or choline-labeled PET ligands [7], and showed substantial impact on management [8]. Moreover, the examination is gaining increasing importance for the initial staging of intermediate and high-risk prostate cancer [9–14].

Several PSMA ligands are available, radiolabeled with currently primary two different positron-emitting isotopes: gallium-68 [^68^ Ga] and fluorine-18 [^18^F].The most commonly used PSMA agent in Europe was initially [^68^ Ga]-PSMA-11, with well-established application for cancer localization in early BCR, with high detection rates, and an impact on management following 60% of scans [15]. In the USA, [^68^ Ga]-PSMA-11 was the first prostate cancer PET tracer to be approved in December 2020 [16]. More recently, [^18^F]-PSMA ligands have become more available, replacing [^68^ Ga]-PSMA ligands. The [^18^F]-labeled DCFPyL showed promising results with high image quality and good lesion detection [17], and similar results were also observed with a new class of radiohybrid tracers (rhPSMA-7) that can be labeled with [^18^F] or metals such as [^68^ Ga], respectively [18]. The major technical advantages of [^18^F]-PSMA ligands over [^68^ Ga]-PSMA ligands are the longer half-life (110 min vs. 68 min) and higher production capacity, as it is produced in cyclotrons rather than generators, resulting in greater availability and fewer logistical challenges. The lower positron emission energy of [^18^F]-PSMA ligands than [^68^ Ga]-PSMA ligands (0.6 MeV vs. 2.3 MeV) also leads to a higher image resolution in comparative phantom studies [19]. One of the candidate ligands already implemented in clinical routines in several hospitals in Switzerland is [^18^F]-PSMA-1007, which benefits from low background activity in the urinary tract [20], which is an important advantage in suspected local recurrence [5, 21].

However, with the greater use of [^18^F]-PSMA-1007, initial studies have reported a higher frequency of unclear focal uptakes than for [^68^ Ga]-PSMA ligands, especially in the lymph nodes, ganglia, and bones [22]. In contrast to the nonspecific uptake in the axillary or mediastinal lymph nodes and the physiologic uptake in the ganglia, unspecific bone uptake (UBU) without morphological correlates might be interpreted as metastasis, with the potential for over-staging the patient, leading to inadequate therapy. The aim of this retrospective multicenter study was to analyze the frequency, anatomical distribution, characteristics, and influencing parameters for UBUs in [^18^F]-PSMA-1007 PET and to evaluate their therapeutic impact.

## Methods

### Study design and population

In this study, we analyzed all [^18^F]-PSMA-1007 scans from four centers (centers A, B, C, and D) obtained between October 2019 and July 2020. This retrospective multicenter study was approved by the lead ethics committee, with general consent present at three centers and waived in one center (EKNZ ID: 2020–01,775). Only patients rejecting the general consent were excluded from the study, which was conducted in compliance with ICH-GCP rules and the Declaration of Helsinki.

Patients with histology-proven prostate cancer of any tumor stage underwent [^18^F]-PSMA-1007-PET due to early BCR, for staging, or for general tumor evaluation (TE). Patient characteristics were collected, including age, initial tumor stage (TNM classification) if available, ISUP score for histological grading [23], bone metastasis, and PSA value less than 4 weeks before the scan. For patients with repeat [^18^F]-PSMA-1007-PET during the study period, only the first scan was included and further imaging used for follow-up analysis.

**Fig. 1:**
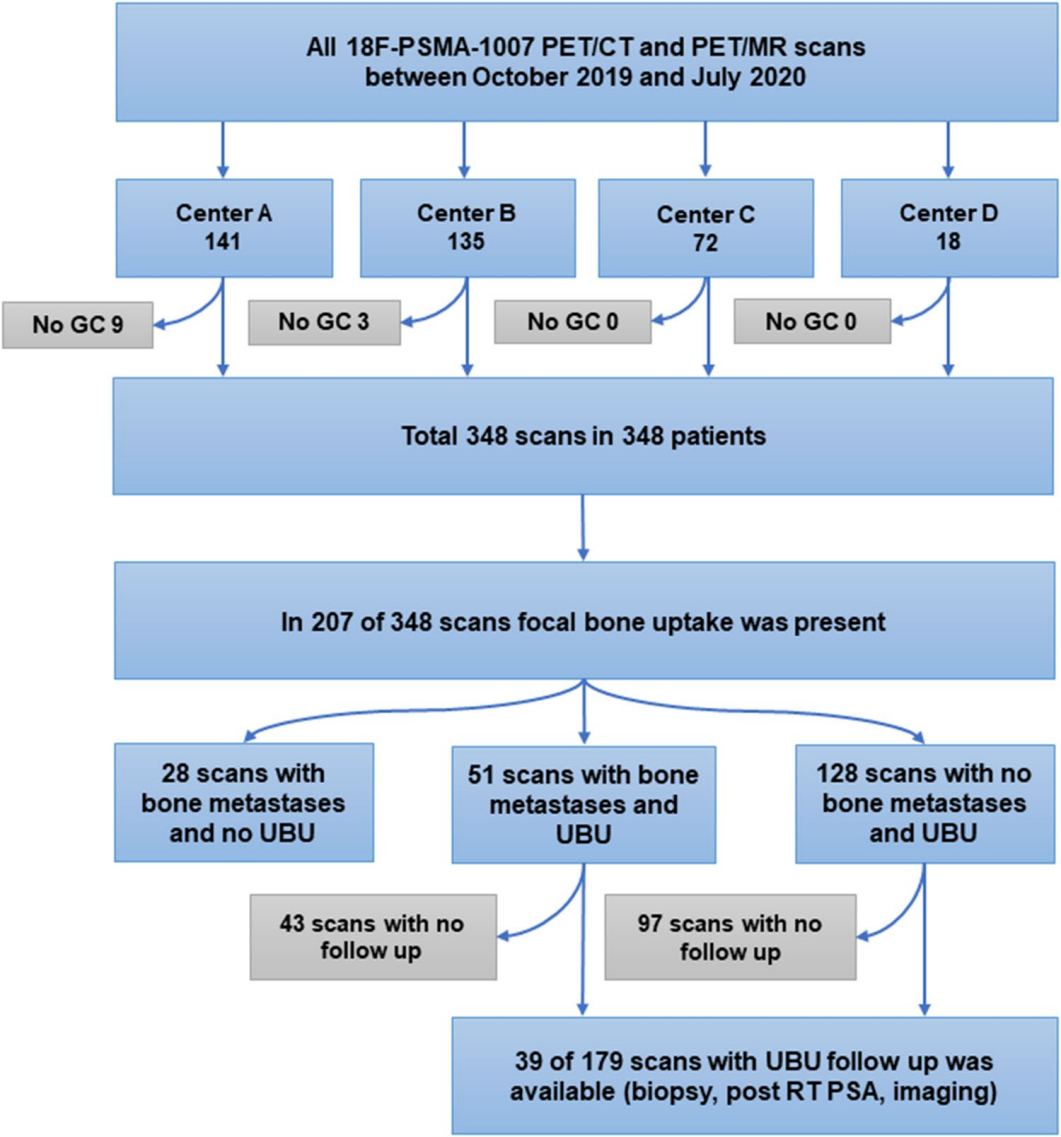
Study flow diagram. RT: radiotherapy

A hybrid PET/CT scanner or a hybrid PET/MR scanner incorporating MR and PET scanners with time of flight was used for the acquisition of the datasets. Imaging was performed using five analog PET/CT scanners (GE PET-CT Discovery 600 and 690, GE Healthcare, Waukesha, WI; Siemens PET/CT Biograph mCT Flow, Siemens Healthineers, Munich, Germany), two digital PET/CT scanners (GE Discovery Molecular Insights – DMI PET/CT, GE Healthcare, Waukesha, WI), and one digital PET/MR with silicon photomultiplier technology (Signa PET/MR, GE Healthcare, Waukesha, WI, USA). At center A, only digital PET scanners were available (two PET/CT and one PET/MR). The injected dose of [^18^F]-PSMA was 3–4 MBq/kg at all the centers, and the uptake time was 60–90 min. The maximal injected dose was not more than 350 MBq. Imaging protocols in detail for all four centers are presented in Supp. 1. The study flow diagram is presented in Fig. 1.

### UBU-based assessment

**Fig. 2:**
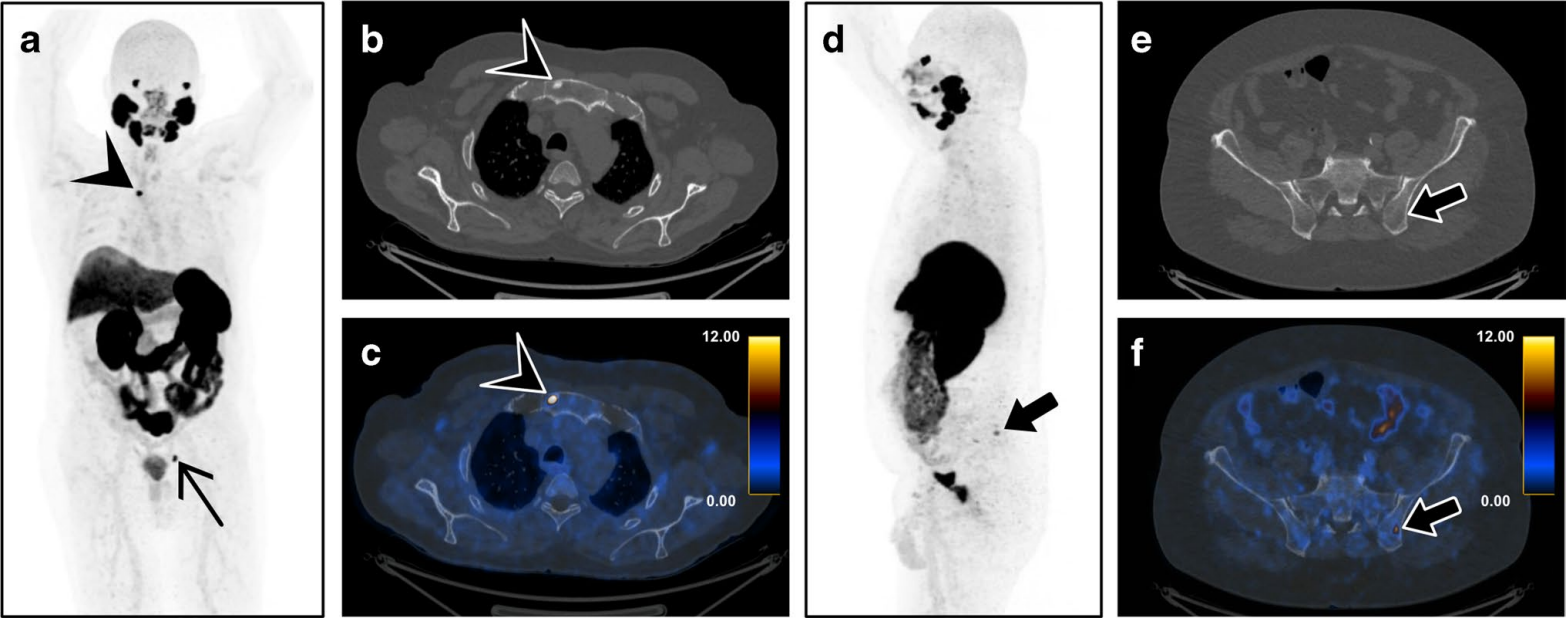
[^18^F]-PSMA-1007-PET/CT on a digital scanner illustrates bone metastasis and UBU in two different patients with prostate cancer. **a** A whole-body maximum intensity projection of PET shows a high PSMA-positive bone lesion (SUV_max_ 23.8) in the manubrium sterni (arrowhead) and a lymph node metastasis in the left pelvis (thin black arrow). **b** Focal sclerosis in the manubrium sterni (arrowhead) on axial CT, **c** corresponding to the high PSMA uptake on the fused images, suggests bone metastasis. **d** A whole-body maximum intensity projection of PET with a moderate PSMA-positive lesion (SUV_max_ 9.5) in the left iliac bone (bold arrow), **e** without morphological correlation on axial CT, was rated as an unspecific finding on **f** PET/CT imaging

All scans were analyzed by one reader with access to clinical information for the presence of UBU, defined as lesions with SUV_max_ below 10 and with neither morphological correlates that suggest metastatic disease nor clear benign findings, such as inflammatory joint diseases, fibrous dysplasia, or fractures. Based on previous observations, bone UBUs have an uptake between SUV_max_ 3 and 10 [24]. If present, the three most active UBUs in each patient were quantified (SUV_max_) and localized (Fig. 2). UBUs were localized in the skull, spine, ribs, sternum, pelvis, and extremities. The datasets were read by physicians who were double board-certified in radiology and nuclear medicine.

### Patient-based assessment

The relationships between the frequency of UBUs and clinical parameters such as age (years), PSA (ng/ml), PSMA uptake time, Gleason score categorized according to the International Society of Urological Pathology (ISUP), with prognostic grade groups 1 to 5, and tumor size (T1 to T4) were analyzed. For patients with UBUs, the therapeutic impact was evaluated: A clinical problem (CP) was considered to be present if the UBUs would alter management when judged as probably benign or malignant—for example, in patients with early BCR with a solitary UBU in the skeleton or in patients referred for staging with UBUs that would prevent local radical therapy if interpreted as malignant. No CP due to UBUs was considered present with either multiple bone metastasis or several clear lesions that had already been excluded from targeted therapy. Furthermore, we retrospectively analyzed whether further clinical investigations were performed, such as additional imaging, biopsy, or PSA value, after radiotherapy. For those patients, the UBUs were classified as malignant, benign, or still unclear based on the follow-up investigation.

### Differences between institutions and scanners

The numbers of patients, numbers of analog and digital PET scanners, frequency of UBUs, and mean SUV_max_ of the UBUs for each center were collected. The relationships between the frequency of UBUs and both the injected dose of [^18^F]-PSMA-1007 and scanner type (analog vs. digital) were analyzed.

### Statistical analysis

Continuous variables were summarized as medians and IQR, and categorical variables were summarized as numbers and percentages. All continuous variables were tested for normal distribution with the D’Agostino–Pearson test, and normality was rejected if *p* < 0.05. Continuous data were compared with the Mann–Whitney test, and the U, Z, and *p* values and 95% CI were calculated and presented in box–whisker plots. Categorical data were compared using chi-squared tests; if significant, Pearson’s contingency coefficient (C) was calculated. Sankey diagrams were used to visualize the clinical impact of UBUs and the outcomes of different follow-up methods. A *p*-value < 0.05 was considered statistically significant in all cases. Statistical analysis was performed using the MedCalc Statistical Software version 19.1 (MedCalc Software bv, Ostend, Belgium). Sankey diagrams were designed with *e!*Sankey 5.2.1 (ifu Institut für Umweltinformatik Hamburg GmbH, Hamburg, Germany).

## Results

### Patient characteristics and demographic data

Patient characteristics and demographic data are shown in Table 1.

**Table 1:** Patient characteristics and demographic data

Number of patients	348
Age (y) (median, IQR)	71.0 (66–76)
Indication for [^18^F]-PSMA-1007-PET, n = 348	
Early BCR	227 (65.2%)
Tumor evaluation	71 (20.7%)
Staging	49 (14.1%)
Initial T classification, n = 281	
T1	30 (10.7%)
T2	88 (31.3%)
T3	151 (53.7%)
T4	12 (4.3%)
Initial N classification, n = 267	
N0	163 (61.0%)
N1	97 (36.3%)
Nx	7 (2.6%)
Initial M classification, n = 267	
M0	215 (83.7%)
M1	23 (8.9%)
Mx	19 (7.4%)
Resection boundaries (R), n = 142	
R0	62 (43.7%)
R1	80 (56.3%)
Patients with bone metastasis	79 (22.7%) of 348
Median (IQR) of PSA values [ng/ml]; n = 306	
Overall	2.5 (0.5–9.3)
Early BCR	1.2 (0.4–4.0)
Tumor evaluation	10.7 (3.3–87.0)
Staging	11.7 (11.7–34.7)
ISUP grade groups, n = 291	
ISUP 1	23 (7.9%)
ISUP 2	48 (16.5%)
ISUP 3	83 (28.5%)
ISUP 4	74 (25.4%)
ISUP 5	63 (21.6%)

Values are given as absolute numbers and percentages in parentheses or median

*BCR* biochemical recurrence; *IQR* interquartile range

### UBU-based assessment

**Fig. 3:**
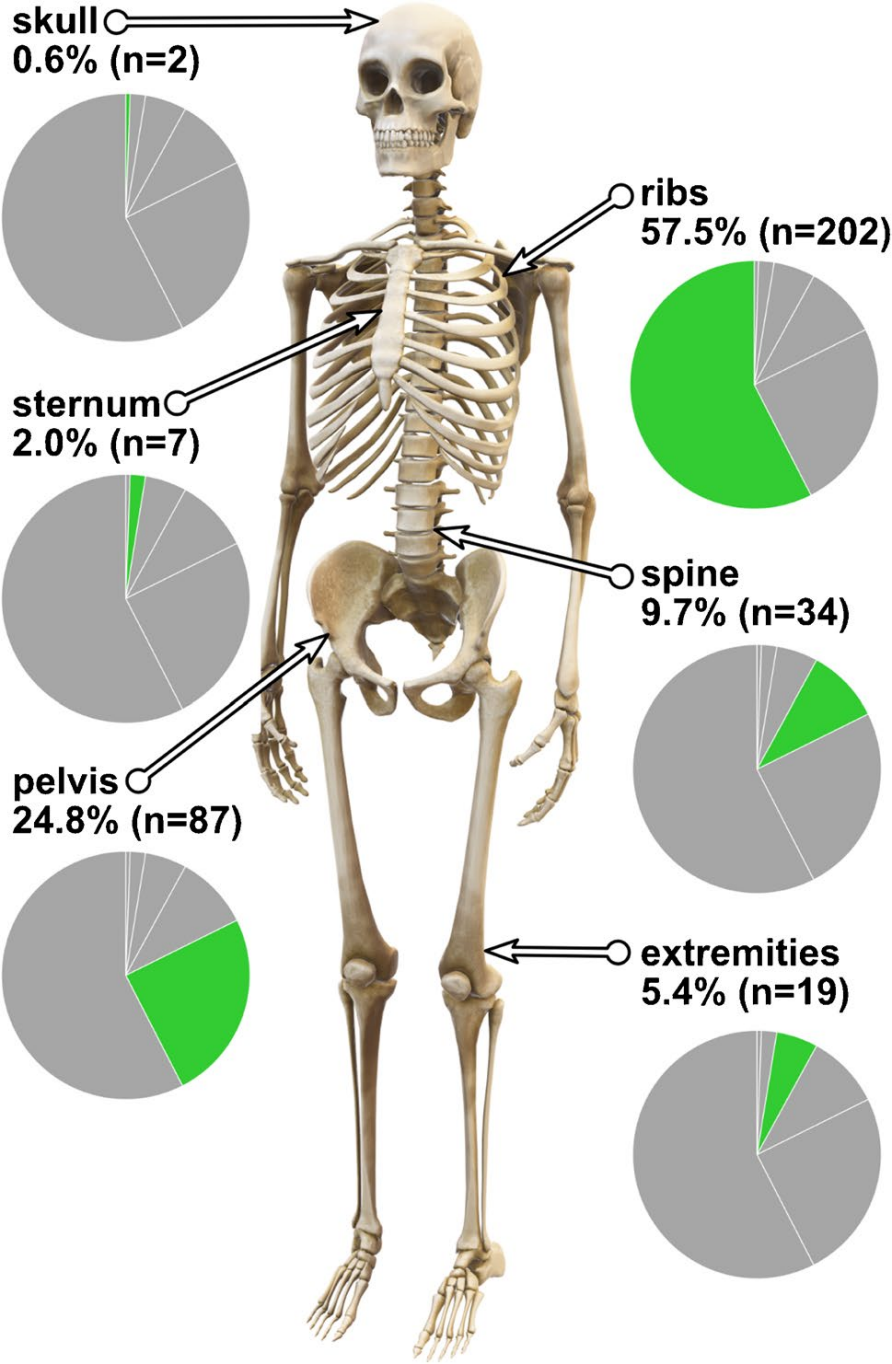
Percentage and number of the three most active UBUs by anatomic region in [^18^F]-PSMA-1007-PET

A total of 348 scans were evaluated, and 351 UBUs were selected in 179 (51.4%) of the patients. Overall the median of UBUs per patient was 2 (IQR 1–4). The most frequent localization was the ribs (57.5%), with a mean SUV_max_ of 3.8, followed by the pelvis (24.8%, SUV_max_ 5.0), spine (9.7%, SUV_max_ 4.7), extremities (5.4%, SUV_max_ 4.6), sternum (2.0%, SUV_max_ 4.3), and skull (0.6%, SUV_max_ 5.9). The mean SUV_max_ of all UBUs was 4.2 ± 2.0 (Fig. 3).

### Patient-based assessment

**Fig. 4:**
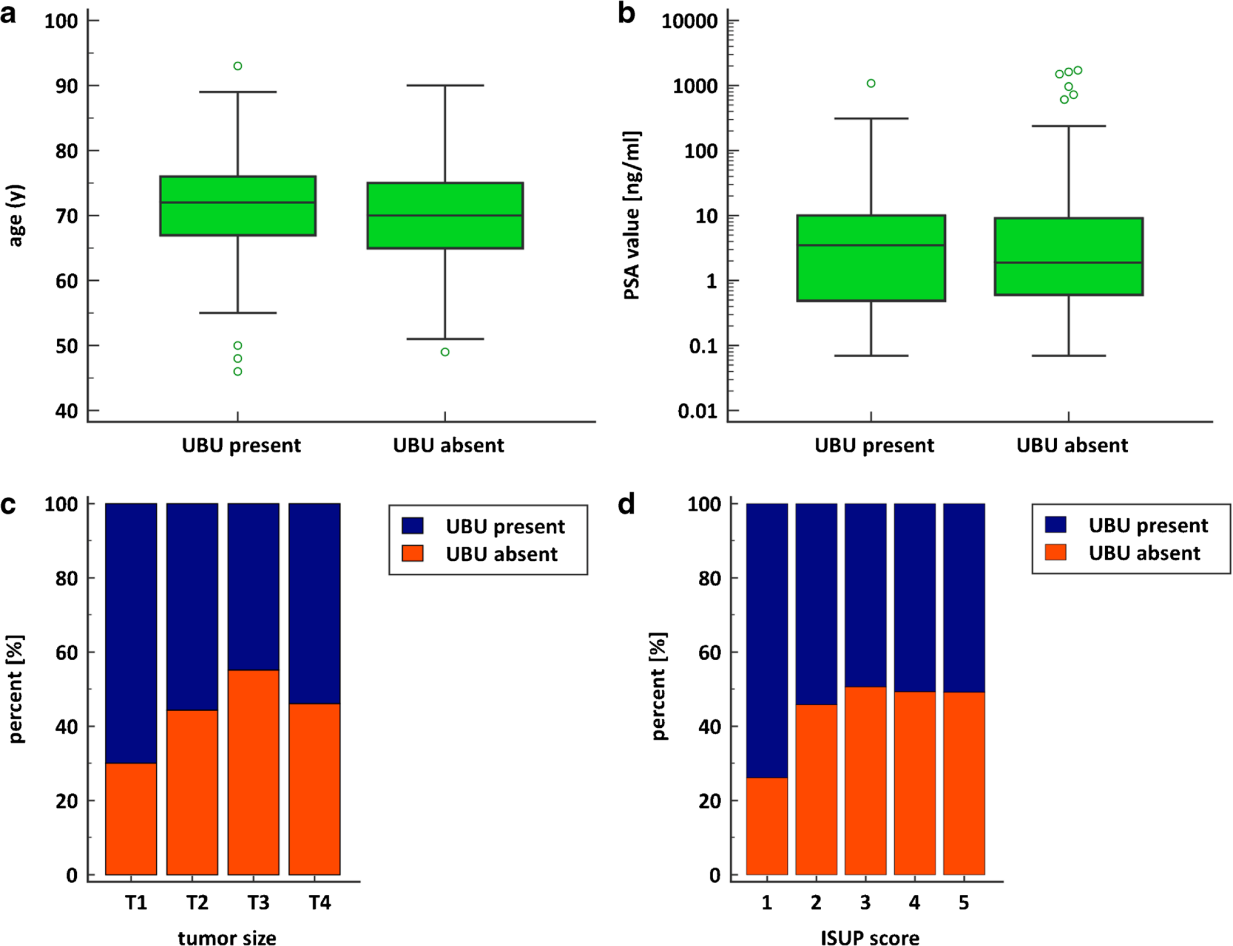
**a** Box–whisker plots show no significant association between age and frequency of UBUs (*p* = .02, but the 95% CI of median difference was not significant). **b** Box–whisker plots show no significant association between PSMA value and the frequency of UBUs (*p* = .05). **c** Bar charts with the percentage distribution of the frequency of UBUs for each tumor size. **d** Bar charts with the percentage distribution of the frequency of UBUs for each ISUP score

Age was not associated with frequency of UBUs (U = 13,014.00, Z = 2.25, *p* = 0.02); although the *p*-value was < 0.05, the 95% CI of median difference was not significant (median difference − 2.0, 95% CI − 3.0 to 0.0) (Fig. 4a). The frequency of UBUs was not associated with PSA value (U = 11,090.00, Z = 0.68, *p* = 0.50) (Fig. 4b). Chi-squared tests showed no relationship between the frequency of UBUs and either tumor size (χ^2^(3) = 5.61, *p* = 0.0573) or ISUP score (χ^2^(4) = 4.78, *p* = 0.3108) (Fig. 4c-d).

**Fig. 5:**
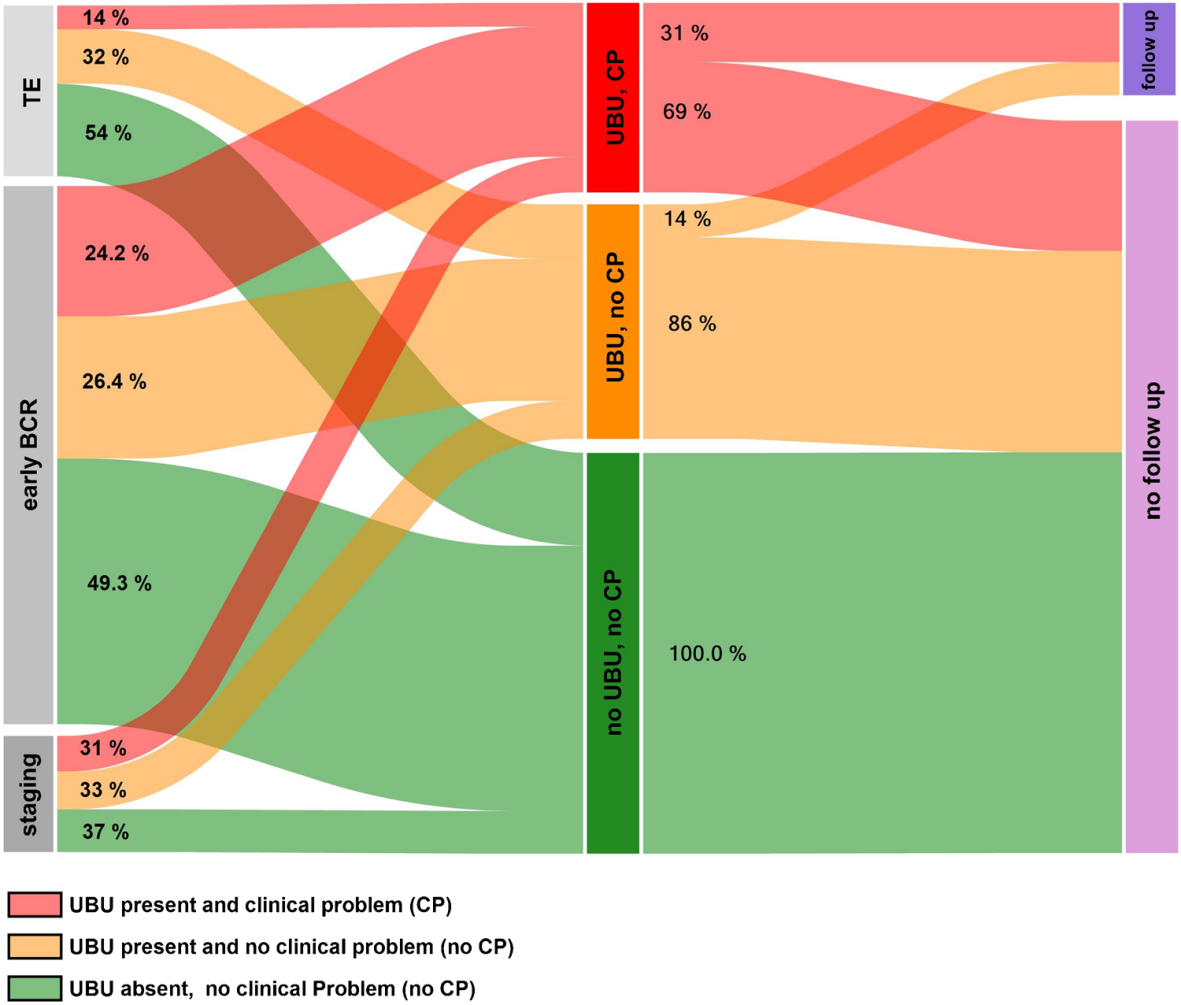
The left part of the Sankey diagram shows the frequencies of patients with UBU-related clinical problems (CP) or without UBU-related CPs for early biochemical recurrence (BCR), tumor evaluation (TE), and tumor staging. The right part of the Sankey diagram shows the percentages of patients with and without follow-up examinations

Overall, in 80 out of 179 patients (44.7%) with UBUs, the lesions were considered a CP that could alter management. Regarding the indication for the PET scan, CPs were present in 55 of 227 patients with early BCR (24.2%), in 15 of 49 for tumor staging (31%), and in 10 of 72 for TE (14%). UBUs with no CPs were present in 60 of 227 (26.4%) patients with early BCR, 16 of 49 for tumor staging (33%), and 23 of 72 for TE (32%). Overall, 39 out of 348 patients (13.8%) were followed by imaging, biopsy, or radiotherapy with PSA follow-up. Only 25 out of 80 patients (31%) with a CP were followed (Fig. 5).

**Fig. 6:**
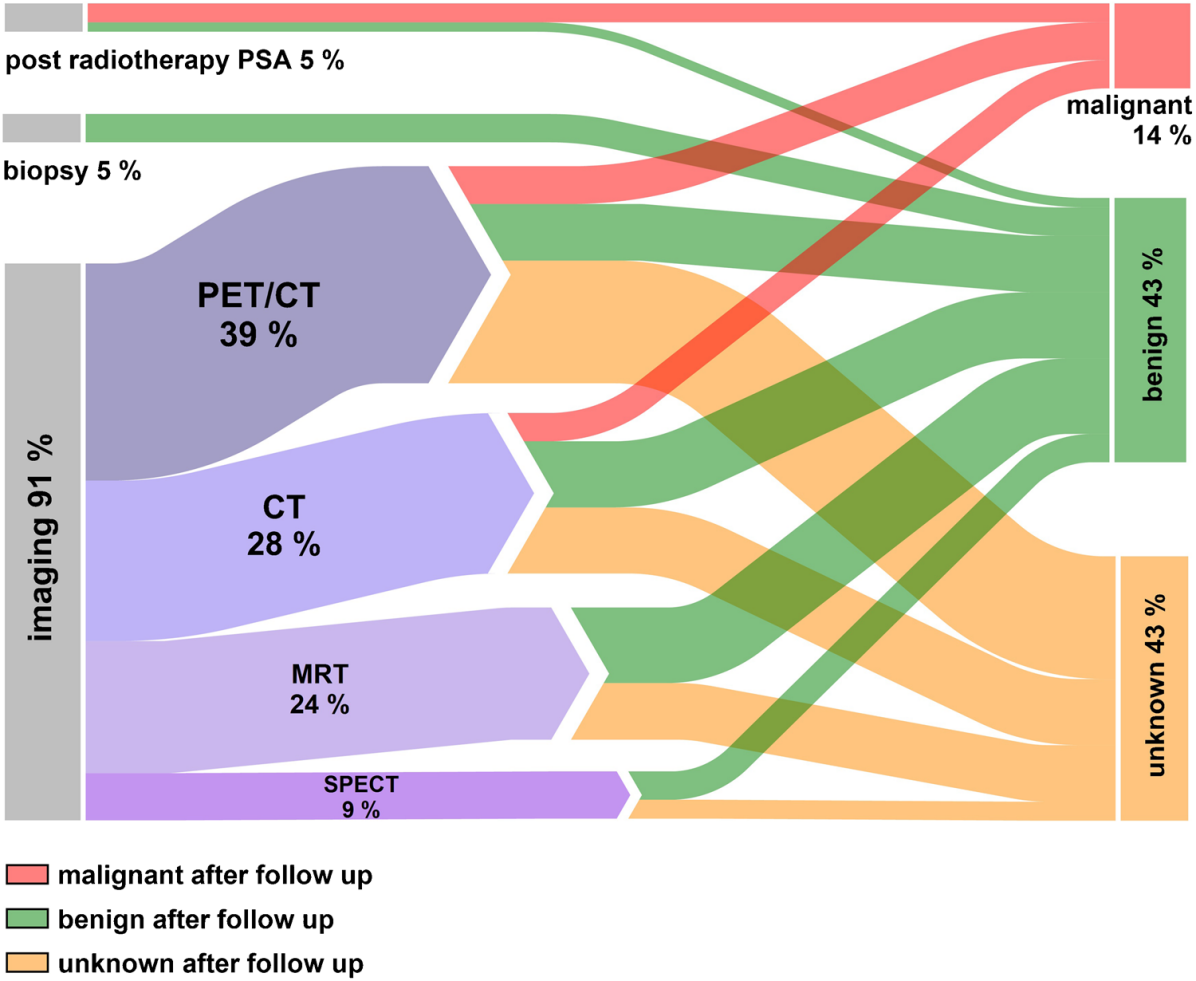
The Sankey diagram shows the percentages of UBUs that were followed up by imaging, biopsy, and post-radiotherapy PSA, as well as the final assessment of the lesions after follow-up (malignant, benign, and unknown)

Follow-up was available for 65 UBUs: three (5%) biopsies (all benign), three (5%) radiotherapies with PSA follow-up (two malignant and one benign) (Table 2), and 59 (91%) imaging follow-ups (seven malignant, 24 benign, 28 unknown). Overall, 59 UBUs were followed up by imaging: 23 (39%) by PET, 17 (29%) by CT, 14 (24%) by MRI, and five (9%) by SPECT. However, after follow-up, 28 (43%) UBUs were still considered unknown, with 28 (43%) benign and nine (14%) malignant (Fig. 6, table provided in Supp. 2). On a scan base, follow-up was available in 39 scans of 179 (21%) with UBUs, and in 41% of the scans, all lesions were considered benign bases on follow-up data (Supp. 3).

**Table 2:** Outcome for biopsy and post-radiotherapy PSA

	SUV_max_	Outcome biopsy and post-radiotherapy PSA
Biopsy		
Pelvis	3.3	Hyperplastic bone marrow
Pelvis	9.5	Paget’s disease
Pelvis	9.9	Hyperplastic bone marrow
Post-radiotherapy PSA		
Rib	2.5	Malignant
Rib	6.8	Benign
Spine	5.7	Malignant

Post-radiotherapy outcome was defined as benign, if PSA level raised after radiation of the UBU. Post-radiotherapy outcome was defined as malignant, if PSA level significantly dropped after radiation of the UBU

### Differences between institutions and scanners

**Fig. 7:**
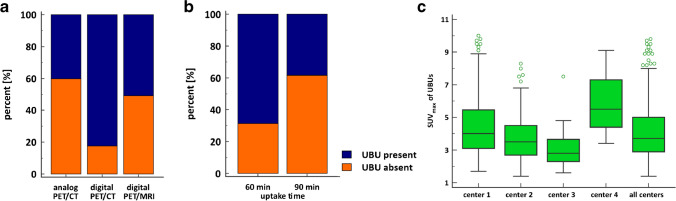
**a** Bar charts with the percentage distribution of the frequency of UBUs for analog PET/CT scanners, digital PET/CT scanners, and digital PET/MRI scanners. **b** Bar charts with the percentage distribution of the frequency of UBUs for PSMA uptake time. **c** Box–whisker plots showing the SUV_max_ for UBUs at each center separately and for all centers combined

Of the 348 PET scans, 132 (37.9%) were performed in center A, 126 (36.2%) in center B, 72 (20.7%) in C, and 18 (5.2%) in D; 221 (63.5%) patients were scanned on an analog PET/CT scanner, 74 (21.3%) on a digital PET/CT scanner, and 53 (15.2%) on a digital PET/MRI scanner. Only in center A were digital PET/CT scanners or PET/MRI scanners available; in center A, only six PET/CT scans were performed on an analog scanner. There were significantly more UBUs present on digital PET scanners (70.1%) than analog scanners (40.7%) (χ^2^(1) = 27.74, *p* = 0.0001, C = 0.27). Subanalysis shows also a higher incidence of UBUs in digital PET/CT compared with analog PET/CT (χ^2^(1) = 14.64, *p* = 0.0001) (Fig. 7a). However, comparison of analog PET/CT with digital PET/MRI shows no difference in the incidence of UBUs (χ^2^(1) = 1.98, *p* = 0.1599) (Fig. 7a). Uptake time of 90 min was associated with a higher incidence of UBU as well (χ^2^(1) = 31.24, *p* < 0.0001) (Fig. 7b).

The number of patients with UBUs at center A was 92 (69.7%), at center B was 50 (39.7%), at center C was 26 (36%), and at center D was 11 (61%) (Supp. 4). The mean SUV_max_ and number of analyzed UBUs at center A were 4.6 and 193 (55.0%), respectively; at center B, they were 3.9 and 98 (27.9%), respectively; at center C, they were 3.0 and 42 (12%), respectively; and at center D, they were 5.8 and 18 (5%), respectively (Fig. 7c). At none of the centers was an association between the occurrence of UBUs and injected activity found (center A: U = 1649.00, Z = 0.95, *p* = 0.34; center B: U = 1806.50, Z =  − 0.47, *p* = 0.64; center C: U = 446.50, Z = 1.78, *p* = 0.08; center D: U = 17.00, Z =  − 1.95, *p* = 0.05) (Supp. 4). The mean and SD for injected activity at center A was 233 ± 40.4 MBq, for center B was 299.4 ± 35.2 MBq, for center C was 282 ± 34.9 MBq, and for center D was 258.8 ± 50.0 MBq (Supp. 5). There was also no association between the occurrence of UBUs and injected activity in digital PET/CT (U = 341.50, Z = 0.45, *p* = 0.66) and digital PET/MRI (U = 283.50, Z = 0.99, *p* = 0.32) (Supp. 6).

## Discussion

Our study showed a high frequency (51.4%) of UBUs in patients undergoing [^18^F]-PSMA-1007 PET, and this number is higher compared to previously reported unspecific findings on [^68^ Ga]-PSMA-11 [21]. The first publication that reported an increased number of benign and unspecific lesions using [^18^F]-PSMA-1007, compared to [^68^ Ga]-PSMA-11, also found a substantially higher number of unclear or likely benign findings in the bones (48% vs. 14.7%), lymph nodes (39.2% vs. 13.7%), and ganglia (66.7% vs. 11.8%) [22]. The lower positron energy with higher spatial resolution and the higher signal-to-background ratio due to the longer half-life of [^18^F] compared to [^68^ Ga] were suggested as possible explanations for this higher incidence [22]. Immunohistochemistry studies have shown that PSMA is not only expressed in prostate tissue, but is also present in inflammatory and neovascular tissue [25, 26]. Activated granulocytes in the bone marrow might therefore also lead to focal bone marrow uptake; therefore, bone marrow islands especially in rips and extremities might be a reason for focal uptake. Furthermore, other bone changes such as fibrous dysplasia or Paget’s disease have been suggested as reason for focal bone uptake [27, 28].

Ultimately, the exact mechanism remains unclear; given that UBUs usually persist in follow-up scans, a morphological correlate seems likely. In our cohort, only three UBUs were biopsied, all of them localized in the pelvis and all diagnosed as benign (two hyperplastic bone marrow and one Paget’s disease).

In our cohort, there was no association between UBU and age, ISUP score, tumor size (T classification), or PSA level, further underlining the difficulty of interpreting the lesions. The lack of correlation between clinical parameters for tumor aggressiveness with UBU presence further supports the hypothesis that these findings are not cancer related. The most common site of UBU was in the ribs, followed by the pelvis and spine. Wang et al. analyzed the distribution of prostate cancer bone metastases based on bone scans [29] and found that patients with a low number of lesions were most likely to have metastases in the spine, followed by the pelvic bones. They also found that only 1% of patients had bone metastasis outside the spine and pelvis without also having metastasis in those regions. This reinforces the theory that singular or multiple UBUs in the ribs without coexisting suspicious lesions in the spine or pelvis are most likely benign. However, although unlikely, there are cases with solitary bone metastasis in the sternum or ribs, as shown in Fig. 2a-c. More sensitive imaging tools than bone scans might also detect more uncommon locations of bone lesions.

Bone metastasis occurs in approximately 10% of patients with newly diagnosed prostate cancer, rising to 80–90% of patients in the advanced stage [30–32]. Therefore, accurate assessment of bone involvement has a direct impact on therapy at every stage in the course of the disease; especially in patients undergoing staging or scanning for early BCR, an M1b situation completely shifts the therapeutic approach from curative to palliative [15].

There was a wide variation of frequencies of UBUs between the different centers, ranging from 36 to 69.7%. This difference might be attributed to the higher scanner sensitivity, given that center A, at 69.7%, was the only institution with digital PET technology. Several studies have shown improvements in lesion detection with digital scanners compared to analog systems for ^18^F-FDG PET, possibly also leading to higher detectability of UBUs using [^18^F]-PSMA-1007 [33, 34]. This was suggested by Alberts et al., who reported a higher detection rate of both prostate cancer lesions and benign PSMA-avid lesions with digital than with analog systems [35]. When comparing digital PET/MR with analog PET/CT, this difference was not visible, and this could be due to the slightly reduced sensitivity of digital PET detectors in MRI scanners due to coils and the magnetic field [36]. It can therefore be assumed that, with more installations of high-end digital PET systems, the frequency of UBU in ^18^F-PSMA-1007 imaging will become even more challenging. We also observed a higher incidence of UBU on scan after 90 min compared to 60-min uptake time, and this could be due to an increased visibility of lesions after 90 min due to reduced background uptake.

**Fig. 8:**
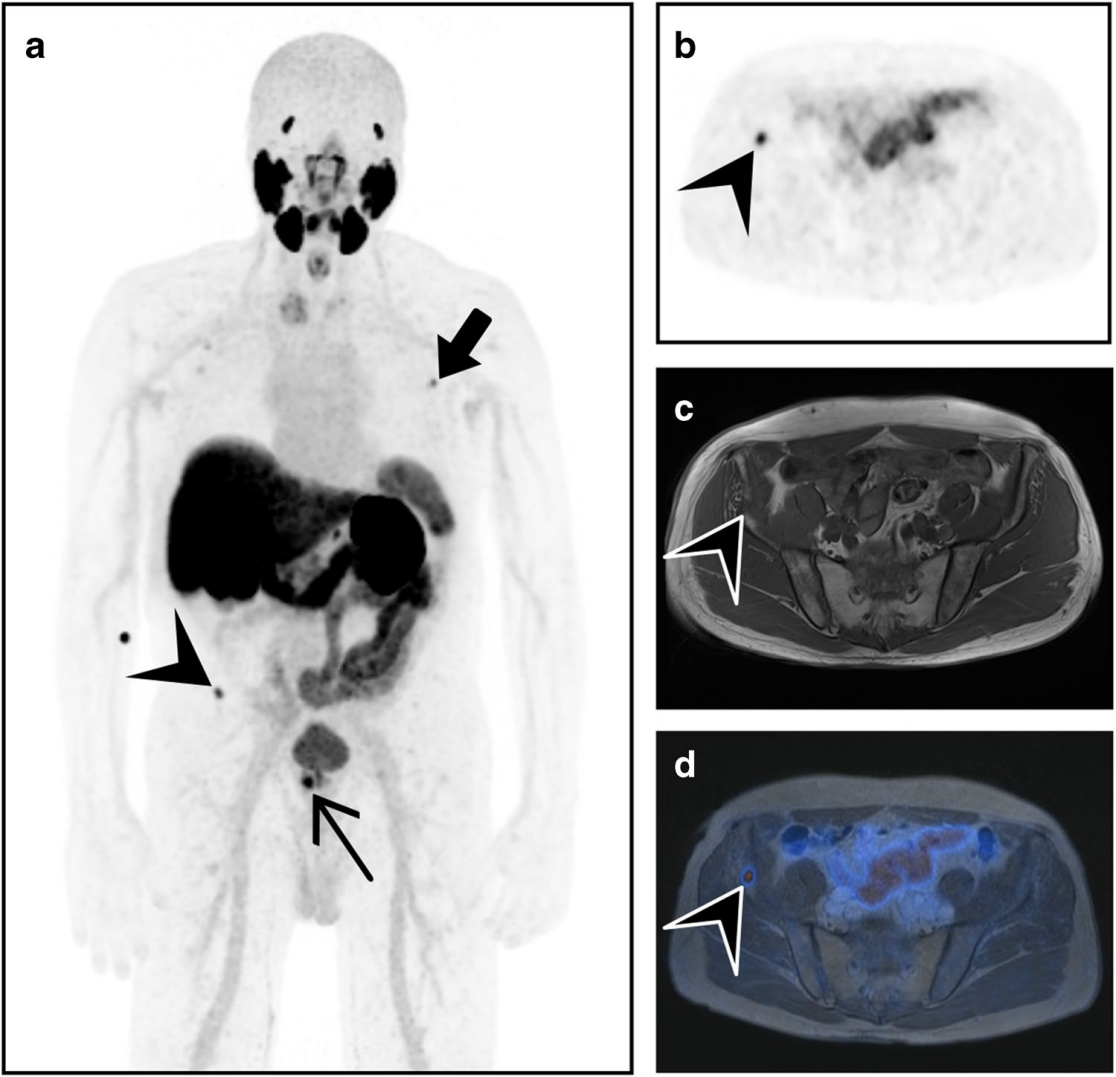
Pretherapeutic [^18^F]-PSMA-1007-PET/MRI staging examination of a 71-year-old patient with prostate cancer, tumor stage pT3b pN0, ISUP 4, and initial PSA of 24.4 µg/l. **a** A whole-body maximum intensity projection showing the PSMA-positive prostate cancer (thin arrow), no lymph node metastasis, an unspecific bone uptake (SUV_max_ 7.0) in one left rib (bold arrow), and a stronger PSMA-positive bone lesion in the right pelvis (SUV_max_ 8.7). **b** The pelvic bone lesion in PET (arrow head). **c** A hypointense morphological correlate on T1-weighted imaging. **d** The lesion on fused PET/MRI (arrow head). After radical prostatectomy, the PSA normalized (several follow-ups with a PSA of < 0.05 µg/l) without further therapy, suggesting that both bone lesions were most likely benign

The increase in UBU in [^18^F]-PSMA-1007 imaging might impair clinical decisions and could lead to an increase in follow-ups with imaging in cases of ambiguity. Due to the significantly higher number of UBUs with [^18^F]-PSMA-1007, the previously suggested standardized image interpretation for [^68^ Ga]-PSMA-PET/CT published by Fanti et al. should not be adopted for [^18^F]-PSMA-1007 [37], or else the frequency of findings defined as pathological could be significantly higher, leading directly to an overdiagnosis of bone metastasis. Figure 8 shows an example of PSMA-avid bone lesions over-diagnosed as bone metastasis in [^18^F]-PSMA-1007 PET/MRI, with a potential impact on the patient’s primary treatment protocol.

The clinical significance of lesions is very subjective and can only be evaluated by incorporating clinical information, as well as the patient’s decision, which was beyond the scope of this retrospective study. We therefore evaluated the hypothetical impact of UBUs on patient management in the different imaging settings and tried to reflect how often UBUs actually resulted in a CP. Based on this assessment, 80 out of 179 patients with UBUs were considered to have CPs (44.7%), but despite this relatively high number, only 39 of the 179 (21.8%) had a follow-up examination. This might be partly due the high number of still-unclear results after follow-up imaging (Fig. 5).

This is a retrospective analysis with associated limitations, most important being the lack of histopathological findings for the bone lesions. Histopathological analysis of UBUs was performed in only three out of 179 patients, showing the difficulty of biopsies for lesions without morphological correlates. Imaging or clinical follow-up after radiotherapy was available only in 36 of 179 scans; therefore, 79% of scans with UBUs did not have follow-up exams, furthermore, while the differences in scanners and protocols at the different centers offer some insights into, for example, the impact of scanner technology on the incidence of UBUs, the asymmetric contributions of the different institutions may have led to a certain bias (e.g., center D having a relatively high number of UBUs (61%) despite analog detectors, but an overall low number of scans). In addition, unspecific bone lesions were selected and clinical implications interpreted subjectively, without a second reading or consensus.

## Conclusion

Despite the abovementioned advantages of [^18^F]-PSMA-1007 over [^68^ Ga]-PSMA-11, we found a high incidence of UBUs—which are clinically challenging—in a significant number of patients. If examinations are performed using digital PET scanners, UBUs are detected more frequently than using analog PET scanners. UBUs should be interpreted carefully to avoid over-staging.

## Supplementary Information

Below is the link to the electronic supplementary material.

Supplementary file1 (DOCX 425 KB)

## Data Availability

The analyzed data may be available from the corresponding author upon reasonable request and with the permission of University Hospital Zurich, University of Zurich, Switzerland.
