# Supplementary material for: Focal unspecific bone uptake on [18F]-PSMA-1007 PET: a multicenter retrospective evaluation of the distribution, frequency, and quantitative parameters of a potential pitfall in prostate cancer imaging
Source: Eur J Nucl Med Mol Imaging. 2021 Jun 13;48(13):4483–94. doi: 10.1007/s00259-021-05424-x (PMC8566387; doi:10.1007/s00259-021-05424-x)
Supplement: Supplementary file 1 — Supplementary file1 (DOCX 425 KB) [file 259_2021_5424_MOESM1_ESM.docx]

**Supplements for:**

**Focal unspecific bone uptake on [^18^F]-PSMA-1007 PET: a multicenter retrospective evaluation of the distribution, frequency, and quantitative parameters of a potential pitfall in prostate cancer imaging**

Hannes Grünig, MD^1, a^, Alexander Maurer, MD^1, a^, Yannick Thali, MD^2^, Zsofia Kovacs^3^,

Klaus Strobel, MD^2^, Irene A. Burger, MD^1, 4, b^, Joachim Müller, MD^3, b^

^1^ Department of Nuclear Medicine, University Hospital Zurich, University of Zurich, Switzerland

^2^ Department of Nuclear Medicine and Radiology, Cantonal Hospital Lucerne, Lucerne, Switzerland

^3^ Department of Radiology and Nuclear Medicine, Cantonal Hospital St. Gallen, St. Gallen, Switzerland

^4^ Department of Nuclear Medicine, Cantonal Hospital Baden, Baden, Switzerland

^a^ Shared first authorship

^b^ Shared last authorship

**Corresponding author:** Irene Burger

Email: irene.burger@ksb.ch

Department of Nuclear Medicine

Kantonsspital Baden

Im Ergel 1

CH-5404 Baden

Switzerland

**Supplement 1:**

| **Supplement 1:** Imaging procedures for all four centers | | |
| --- | --- | --- |
|  | Administered PSMA dose | Uptake-time |
| *Center A* | *3.0 MBq/kg* | *60 min* |
| *Center B* | *3.5 MBq/kg* | *90 min* |
| *Center C* | *3.5 MBq/kg* | *60 min* |
| *Center D* | *3.0 MBq/kg* | *90 min* |
|  | |  |

**Supplement 2:**

| **Supplement 2:** Summary and overview of outcome follow up after imaging, biopsy and post-radiotherapy PSA sorted by location. | | |
| --- | --- | --- |
| **Ribs (n=33)** |  |  |
| *SUV_max_ UBU* | *follow up* | *outcome* |
| 2.8 | CT | begnin |
| 6.8 | post-radiotherapy PSA | benign |
| 1.9 | PSMA-PET | benign |
| 2.3 | CT | benign |
| 2.9 | CT | benign |
| 5.0 | PSMA-PET | benign |
| 4.9 | SPECT | benign |
| 1.7 | PSMA-PET | benign |
| 1.7 | PSMA-PET | benign |
| 2.2 | CT | benign |
| 3.7 | PSMA-PET | benign |
| 1.5 | PSMA-PET | benign |
| 1.7 | CT | benign |
| 4.6 | CT | malignant |
| 6.1 | PSMA-PET | malignant |
| 2.5 | post-radiotherapy PSA | malignant |
| 2.4 | CT | malignant |
| 4.3 | PSMA-PET | malignant |
| 2.5 | CT | malignant |
| 6.8 | MRI | unknown |
| 3.7 | SPECT | unknown |
| 2.5 | PSMA-PET | unknown |
| 3.5 | PSMA-PET | unknown |
| 6.5 | FDG-PET | unknown |
| 3.1 | PSMA-PET | unknown |
| 3.1 | MRI | unknown |
| 3.3 | PSMA-PET | unknown |
| 2.9 | PSMA-PET | unknown |
| 4.0 | CT | unknown |
| 4.9 | PSMA-PET | unknown |
| 5.9 | FDG-PET/CT | unknown |
| 3.6 | PSMA-PET | unknown |
| 2.2 | PSMA-PET | unknown |
| **Pelvis (n=20)** |  |  |
| *SUV_max_ UBU* | *follow up* | *outcome* |
| 3.3 | biopsy | benign |
| 9.5 | biopsy | benign |
| 9.9 | biopsy | benign |
| 5.9 | SPECT | benign |
| 3.3 | MRI | benign |
| 3.6 | MRI | benign |
| 3.2 | MRI | benign |
| 3.9 | MRI | benign |
| 2.6 | MRI | benign |
| 3.2 | CT | benign |
| 3.4 | MRI | benign |
| 3.1 | MRI | benign |
| 4.1 | CT | malignant |
| 3.5 | CT | malignant |
| 4.3 | PSMA-PET | unknown |
| 3.7 | MRI | unknown |
| 2.7 | MRI | unknown |
| 3.9 | MRI | unknown |
| 3.5 | CT | unknown |
| 7.0 | CT | unknown |
| **Spine (n=7)** |  |  |
| *SUV_max_ UBU* | *follow up* | *outcome* |
| 4.8 | MRI | benign |
| 4.7 | CT | benign |
| 5.7 | post-radiotherapy PSA | malignant |
| 7.5 | SPECT | unknown |
| 2.3 | PSMA-PET | unknown |
| 7.5 | CT | unknown |
| 7.6 | MRI | unknown |
| **Extremities (n=3)** |  |  |
| *SUV_max_ UBU* | *follow up* | *outcome* |
| 8.3 | SPECT | benign |
| 3.2 | PSMA-PET | unknown |
| 5.6 | FDG-PET | unknown |
| **Sternum (n=2)** |  |  |
| *SUV_max_ UBU* | *follow up* | *outcome* |
| 7.6 | CT | unknown |
| 4.2 | PSMA-PET | unknown |

**Supplement 3:**

Overview of lesion and scan based number of UBUs: If multiple lesions with different outcomes were present, scans were classified according to the dominant finding: Malignant > unknown > benign.

|  | **Lesion based** | | | | | **Scan based** | |
| --- | --- | --- | --- | --- | --- | --- | --- |
|  | UBU 1 (N) | UBU 2 (N) | UBU 3 (N) | Total (N) | % | scans (N) | % |
| malignant | 6 | 3 | 0 | 9 | 14 | 7 | 18 |
| benign | 16 | 8 | 4 | 28 | 43 | 16 | 41 |
| unknown | 17 | 7 | 4 | 28 | 43 | 16 | 41 |
| UBU (N) | 39 | 18 | 8 | 65 | 100 | 39 | 100 |

*N = number*

**
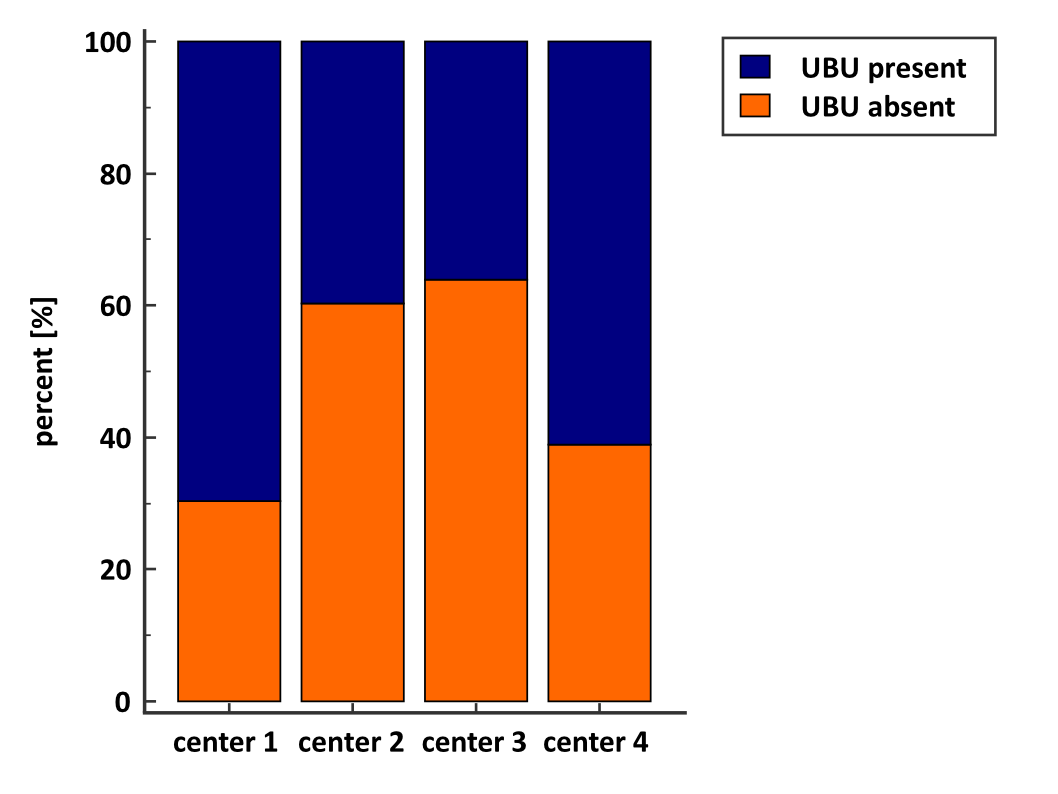
**

**Supplement 4:** Bar charts with percentage distributions of the frequencies of UBUs at each center.


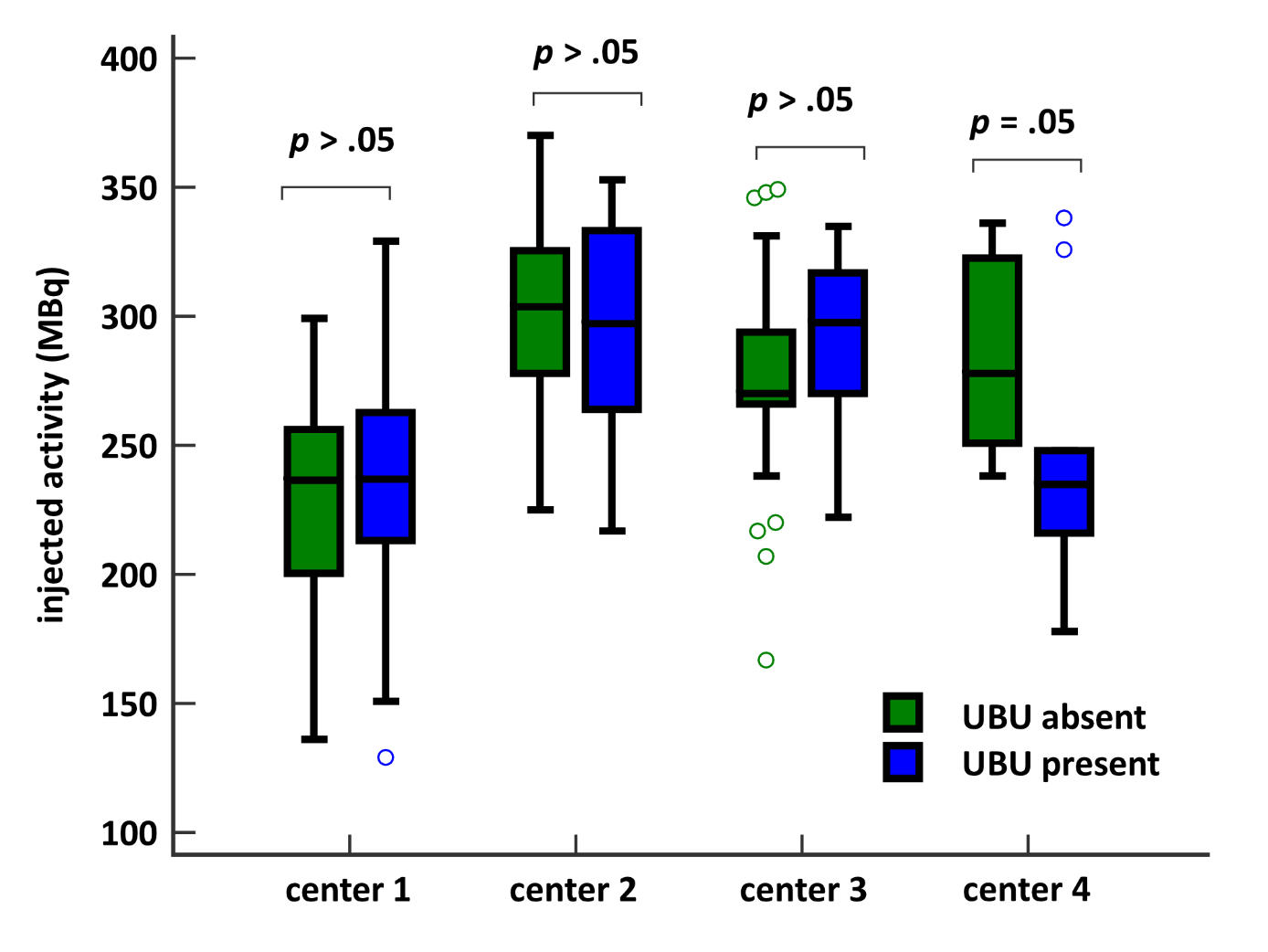


**Supplement 5:** Box–whisker plots show no significant association between injection activity and the frequency of UBUs at each center.


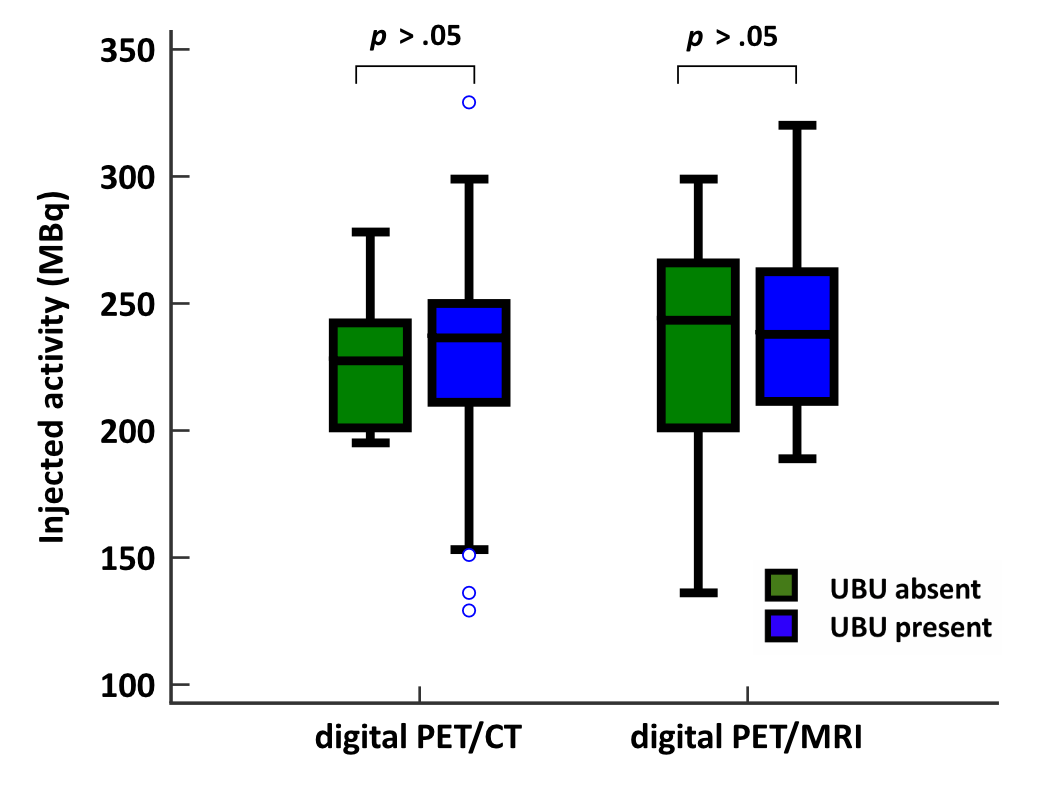


**Supplement 6:** Box–whisker plots show no significant association between injection activity and the frequency of UBUs in digital PET/CT and digital PET/MRI.
